# Nociception level index as a tool of measuring pain objectively in patients with complex regional pain syndrome: a feasibility study

**DOI:** 10.3389/fpain.2025.1669794

**Published:** 2025-12-03

**Authors:** Ibrahim Umar, Avraham Tenenbaum, Arik Tzour, Amran Khalaia, Michael Grach

**Affiliations:** 1 Lady Davis Carmel Medical Center, Haifa, Israel; 2 Medasense Biometrics Ltd., Ramat Gan, Israel

**Keywords:** nociception level index, complex regional pain syndrome, objective pain assessment, chronic pain management, nociception, nociception device

## Abstract

**Introduction:**

This study evaluates the feasibility of using the Nociception Level Index (NOL) as an objective tool for assessing pain in awake patients with Complex Regional Pain Syndrome (CRPS). Traditional pain assessment methods, such as the Visual Analog Scale (VAS), are subjective and influenced by psychological and cognitive factors, limiting their accuracy in chronic pain conditions.

**Methods:**

A single-center, prospective observational study was conducted with 26 CRPS patients. The NOL index, a multi-parameter device measuring autonomic nervous system responses, was assessed alongside VAS scores during physiotherapy exercises and intravenous lidocaine treatment. Pain levels were recorded at baseline, during painful exercises, and post-lidocaine administration. Changes in NOL and VAS scores were analyzed using the Wilcoxon signed-rank test with Bonferroni correction. Receiver operating characteristic (ROC) analysis with bootstrapped 95% confidence interval (CI) evaluated the sensitivity and specificity of NOL and VAS in discriminating painful from non-painful states.

**Results:**

Physiotherapy exercises significantly increased both NOL (from 22.7 ± 10.7 to 30.3 ± 8.9) and VAS scores (from 5.5 ± 2.8 to 8.15 ± 1.6) (*p* < 0.001 for both). Lidocaine treatment led to partial pain relief, with VAS scores decreasing statistically significantly (from 7.5 ± 2.2 to 4.7 ± 2.8, *p* < 0.001), while NOL values trended lower (from 35.3 ± 12.2 to 29.4 ± 15.6, *p* = 0.26). ROC analysis showed comparable discrimination between painful and non-painful states for both tools [AUC: NOL 0.76 95% CI (0.68,0.84); VAS 0.78 95% CI (0.7,0.85)].

**Conclusions:**

The NOL index shows promise as a complementary tool for objective pain assessment in CRPS, potentially offering insights into pain dynamics that subjective tools may miss. Further research with larger cohorts and standardized protocols is recommended to validate its clinical utility in chronic pain management.

## Introduction

Accurate pain assessment is a crucial aspect of effective pain management, essential for guiding treatment decisions and monitoring patient outcomes. For clinicians, reliable pain measurement tools are essential in developing appropriate treatment plans and evaluating therapeutic efficacy. However, traditional pain assessment methods, particularly self-reporting tools, present several limitations, especially in chronic pain populations.

The Visual Analog Scale (VAS) is a widely utilized tool for assessing pain intensity. It requires patients to self-report their pain level on a continuum, typically a 10-centimeter line with the endpoints representing the extremities of pain/no pain. Despite its widespread use, the VAS is not without drawbacks. Emotional factors, such as anxiety and depression, as well as cognitive impairments, can significantly influence patients' pain perception and their ability to accurately report pain intensity. These factors contribute to variability and potential inaccuracies in pain assessment, particularly in chronic pain populations (1).

In the context of CRPS, accurate pain assessment is crucial for effective management and monitoring of disease progression. However, the limitations inherent in self-reporting tools like the VAS are particularly pronounced in CRPS patients, who often experience significant emotional distress and cognitive impairments that can skew pain reporting (2, 3). These challenges underscore the need for objective pain assessment tools in managing CRPS.

To address these limitations, the multi-parameter NOL (Nociception Level) index (Medasense Biometrics Ltd., Ramat Gan, Israel) was developed as an objective tool for monitoring nociception in surgical patients under general anesthesia. This FDA-authorized device integrates various physiological parameters, including heart rate, heart rate variability, photo-plethysmograph, skin conductance, and their time derivatives to generate a single, personalized index reflecting nociceptive responses. The physiological data is captured continuously using a non-invasive finger probe, and an AI machine learning algorithm processes the data to output the NOL index as a numeric value and trend over time, updated every 5 s. While the NOL index was initially developed, validated, and FDA-authorized for use in patients under general anesthesia, this feasibility study aims to explore its potential application in awake patients. By studying the technology's efficacy in conscious individuals, we seek to expand our understanding of objective pain assessment tools and their potential benefits across various clinical settings.

The NOL index is displayed on a scale from 0 to 100, where 0 represents no pain and 100 represents extreme pain. Numerous studies have demonstrated the NOL index's efficacy in accurately detecting nociception and distinguishing between different painful stimuli intensities across various clinical settings (4, 5).

While the NOL index was originally designed and validated for use in anesthetized patients, only a few studies have studied its potential in awake patients (4, 5). Our study contributes to this emerging body of knowledge by examining the NOL index in awake Complex Regional Pain Syndrome (CRPS) patients.

CRPS is a progressive and debilitating condition primarily affecting extremities. It is characterized by continuous pain that is disproportionate to the initial trauma. The pathophysiology of CRPS involves a multifactorial process, including both peripheral and central nervous system mechanisms, with inflammation playing a pivotal role (2). Beyond intense pain, patients with CRPS report a range of symptoms, including limb-specific hypersensitivity, swelling, temperature and color changes, movement difficulties, and a sense of disconnection from the affected limb (3). Diagnosis of CRPS is based on these defining characteristics (6).

The NOL index presents a significant potential for improving pain assessment in CRPS. Although its efficacy has been established as nociception monitoring under general anesthesia, its use in chronic pain conditions like CRPS remains largely unstudied. To date, only a single pilot study, involving a sample size of 20 participants, has evaluated the validity of the NOL index in CRPS patients (7).

This study assesses the potential of the NOL index as an objective and potentially superior tool compared to VAS for pain management in patients diagnosed with neuropathic pain syndrome. We hypothesize that NOL will track dynamic pain response during physiotherapy and lidocaine treatment, reflecting changes more precisely than subjective reports (VAS). By measuring NOL before and after physiotherapy and at key points during lidocaine treatment, while comparing these to VAS scores, this study seeks to establish NOL as an objective tool for the assessment of pain and effectiveness of physical therapy in neuropathic pain patients.

## Methods

This is a single-center, prospective, descriptive observational study conducted at the Pain Unit of the Carmel Medical Center, Haifa, Israel. The study was carried out under the conditions of routine clinical practice, without modifications to the prescribing patterns of the investigators. The study protocol was approved by the Carmel Medical Center Institutional Review Board (0054-21-CMC).

### Study population

Twenty-six (26) participants of both sexes who met the selection criteria were enrolled between 2022 and 2023. The inclusion criteria were age ≥18 years, being diagnosed with peripheral neuropathic pain syndrome for at least 6 months, to ensure chronicity and stability of symptoms, the ability to tolerate a 2-h intravenous (IV) infusion of lidocaine medication at a dose of 1.0 mg/kg/hr., as part of the standard of care in Carmel Medical Center, and having signed the informed consent. To maximize recruitment feasibility, exclusion criteria were intentionally limited and included only criteria were bilateral/generalized neuropathic pain, patients requiring sedation, patients with movement disorders preventing stillness, weight outside the 50–90 kg range, tremor, lidocaine allergy.

All participants underwent continuous monitoring of vital signs throughout the study. Non-invasive blood pressure cuffs were used to measure systolic and diastolic blood pressure, while pulse oximetry provided real-time data on oxygen saturation (SpO2) and electrocardiogram (ECG) was monitoring heart electrical activity. The goal of the continuous monitoring was to ensure patient safety by detecting any potential adverse events associated with the lidocaine procedure. The continuous monitoring data confirmed that no patients experienced adverse events during the study protocol.

For objective pain assessment, the NOL index was utilized. Importantly, the NOL was blinded to the patients involved in the study. This blinding approach was implemented to minimize potential bias in pain assessment. By ensuring that the patients were unaware of the NOL readings, the physician aimed to maintain the integrity of the data collected during physical therapy sessions and subsequent analysis.

The physicians, on the other hand, could read the NOL index. This allowed them to incorporate this objective measure into their clinical observations and decision-making process while managing patient care.

### Study design

**Figure 1:**
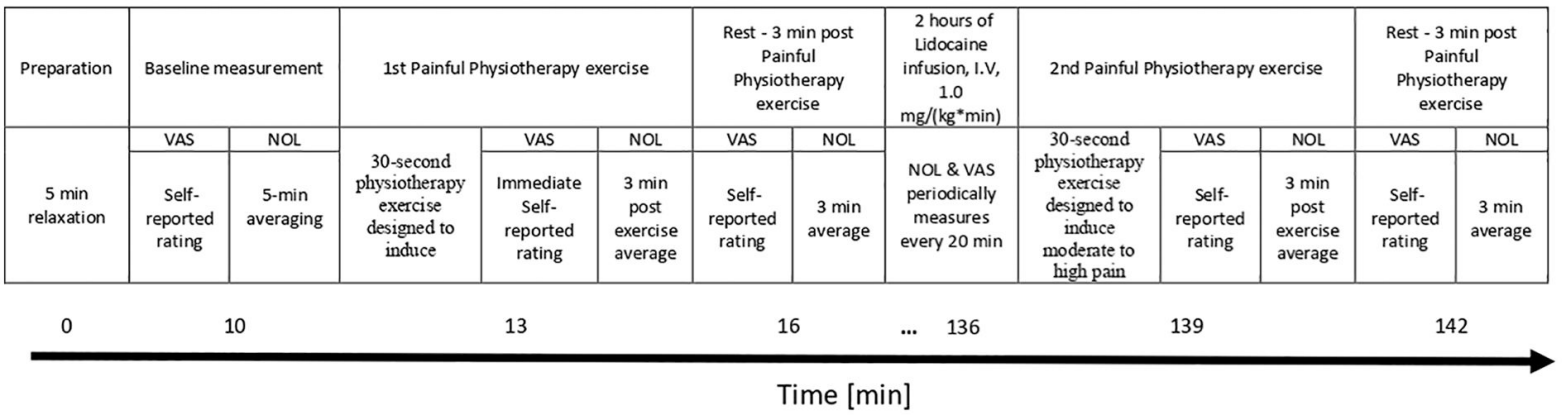
Study design timeline. NOL, nociception level index; VAS, Visual Analog Scale; I.V, Intravenous).

NOL index recording was continuous throughout the entire experiment and averaged at four distinct time points (Figure 1):

1. Baseline: Measured at rest before any interventions. To prepare for the study, participants relaxed in a quiet room for 5 min while NOL-index was recorded continuously. Following this rest, the physician measured their average baseline NOL-index over a period of 5 min. Participants then rated their affected limb's pain intensity using the VAS (rest, pre-physiotherapy, pre-treatment).
2. During Painful Physiotherapy Exercise: Measured during a physiotherapy exercise that causes pain.). The participants performed a 30-second physiotherapy exercise designed to induce moderate to high pain in the affected limb. Each patient was asked by the physician to actively move their limb in a way that stimulates pain, e.g., for affected upper limb, forcefully opening and closing a fist in a manner that was challenging but still manageable for the entire duration. The physician measured their averaged NOL-index over a period of 3 min after starting the physiotherapy exercise. Finally, participants used the VAS to rate their maximum pain intensity experienced during the exercise (pre-treatment, painful- physiotherapy).
3. Rest, Post-Physiotherapy, Pre-Treatment: The participants relaxed for another 3 min, during which the physician measured their averaged NOL-index. Participants then rated their affected limb pain intensity using the VAS (2nd rest, post- physiotherapy, pre-treatment).
4. Rest, Lidocaine Treatment: Following the period of rest, patients received lidocaine treatment (1.0 mg/kg/hr.) via continuous intravenous infusion for two hours. Throughout the treatment, NOL index and VAS assessments were performed every 20 min to monitor pain levels objectively and subjectively, respectively (rest, treatment).
5. Painful Physiotherapy Exercise (Post-lidocaine): Measured during the same Painful Physiotherapy Exercise for 30 s using the affected limb: The physician measured patient's average NOL-index over a period of 3 min after starting the physiotherapy exercise. Upon completion of this period, patients were asked to rate the maximum pain intensity they experienced during the exercise using the VAS (post-treatment, painful physiotherapy).
6. Rest, Post-Physiotherapy, Post-Treatment: The participants relaxed for another 3 min, during which the physician measured their average NOL-index. Participants then rated their affected limb pain intensity using the VAS (3rd rest, post- physiotherapy, post-treatment).

### Lidocaine IV

As part of the department's protocol for the first treatment of each patient, lidocaine was administered at a total dose of 2 mg/kg as an intravenous infusion over 2 h, recognized as an initial dose according to standard dosing guidelines (8). This dosage was specifically chosen and used in accordance with local policy aimed at minimizing potential side effects in at risk populations. All participants were observed closely, and a physician was immediately available over the 2-hour infusion. Patients were educated on common lidocaine side effects, signs of a serious reaction, and instructed to rate their pain (VAS score). Based upon patient response and tolerance of the study infusion, the patient and physician chose to continue or discontinue the study protocol. Data collected from each patient's medical record included dates of treatment, indication, type of infusion, dose, infusion time, adverse events, action taken as a result of an adverse event, duration of treatment, reason for discontinuation, and pain scores.

### Self-reporting pain scale

VAS is based on self-reported measures of symptoms that are recorded with a single handwritten mark placed at one point along the length of a 10-cm line that represents a continuum between the two ends of the scale- “no pain” on the left end (0 cm) of the scale and the “worst pain” on the right end of the scale (10 cm). Measurements from the starting point (left end) of the scale to the patients’ marks are recorded in centimeters and are interpreted as their pain. The values can be used to track pain progression for a patient or to compare pain between patients with similar conditions.

### Missing data

In the analysis, data from all 26 participants were included for the first physiotherapy exercise. However, for the second stimulus, data from 7 patients were excluded due to preterm data collection termination and technical issues. These exclusions were made because missing data on the second physiotherapy exercise could not be reliably imputed or extrapolated without introducing bias. By excluding only the second physiotherapy exercise data from these participants, we maintained the integrity of the complete dataset from the first physiotherapy exercise, allowing for a full baseline analysis while ensuring consistency in data quality for the second stimulus.

### Statistical analysis

Given the feasibility nature of the study, a formal sample size calculation was not performed. Data collected from the 26 enrolled participants were de-identified and transferred to a secure computer database for statistical analysis using python package SciPy version 1.10.1 and MedCalc® Statistical Software (MedCalc Software Ltd, Ostend, Belgium). Initial assessments contradicted a normal distribution; hence a non-parametric approach was used in the analysis of all continuous data. The primary outcome, investigating NOL index variation before and after the physiotherapy exercise, was analyzed using a Wilcoxon signed-rank test. This same statistical approach was employed to evaluate changes in NOL variation with physiotherapy exercise following the lidocaine and VAS variation with physiotherapy exercise in both the pre-and-post-lidocaine treatment. A Bonferroni correction was applied to adjust for multiple testing across eight pairwise comparisons, resulting in an adjusted significance threshold of *α* = 0.05/8 = 0.00625. To evaluate the ability of both VAS and NOL to discriminate between painful stimulus, i.e., physiotherapy exercise, and non-painful stimulus or absence of stimulus, the area under the curve (AUC) of the receiver operating characteristic curve (ROC) was calculated. Corresponding optimal NOL and VAS values were calculated using Youden's J statistic. Statistical significance was established at a *p*-value threshold of equal or less than 0.05. 95% confidence interval (CI) of median values, and AUC ROC was calculated using bootstrap with 1,000 repetitions.

## Results

### Clinical and pain characteristics

A total of 26 individuals with CRPS [11 (42%) females and 15 (58%) males] with a mean age of 42 ± 12.82 participated in the study. Data collection for the second physiotherapy exercise was terminated preterm in 7 participants, resulting in incomplete records for this phase. Consequently, these participants' data were excluded from analysis for the second physiotherapy exercise to ensure accuracy and consistency within the dataset. Individuals with CRPS in this study had varying pain durations, with 4 participants experiencing symptoms for less than 12 months, 5 participants for 12–24 months, and the majority, 17 participants, for over 36 months. The participants have a mean baseline pain intensity of VAS 5.5 ± 2.8. All clinical and pain characteristics are presented in Table 1.

**Table 1:** Baseline demographic data.

Demographic and clinical data of the participants (*n* = 26)		
Sex, *n* (%)	Men	15 (58)
	Women	11 (42)
Age (y), Median (Q1 -Q3)		42.5 (30–50)
BMI (kg/m^2^), mean (STD)		27.16 (5.56)
CRPS-affected extremity, *n* (%)	Upper	13 (50)
	Lower	12 (46)
	Multiple limbs	1 (4)
Time since diagnosis, n, (%)	<12 months	4 (16)
	12–24 months	5 (19)
	>36 months	17 (65)
VAS score of CRPS pain on the day of testing		5.46 (2.77)
Medication, *n* (%)		
Non-Opioid analgesics		17 (65)
	NSAID's Metamizole Paracetamol	1 (4) 6 (23) 13 (50)
Opioids analgesics	Oxycodone Buprenorphine Tramadol Codeine	16 (62) 3 (12) 1 (4) 8 (31) 11 (42)
Anticonvulsant	Gabapentin Pregabalin	8 (31) 5 (19) 4 (15)
Antidepressants	SNRI TCA	7 (27) 4 (15) 3 (12)
Cannabis		7 (27)
Nerve block injections		1 (4)

BMI, body mass index; CRPS, complex regional pain syndrome; NSAID, non-steroidal anti-inflammatory drugs; Q, quartile; SNRI, serotonin-norepinephrine reuptake inhibitors; TCA, Tricyclic antidepressant; VAS, visual analogue scale.

### Pre-Lidocaine treatment: pre and post physiotherapy exercise

**Figure 2:**
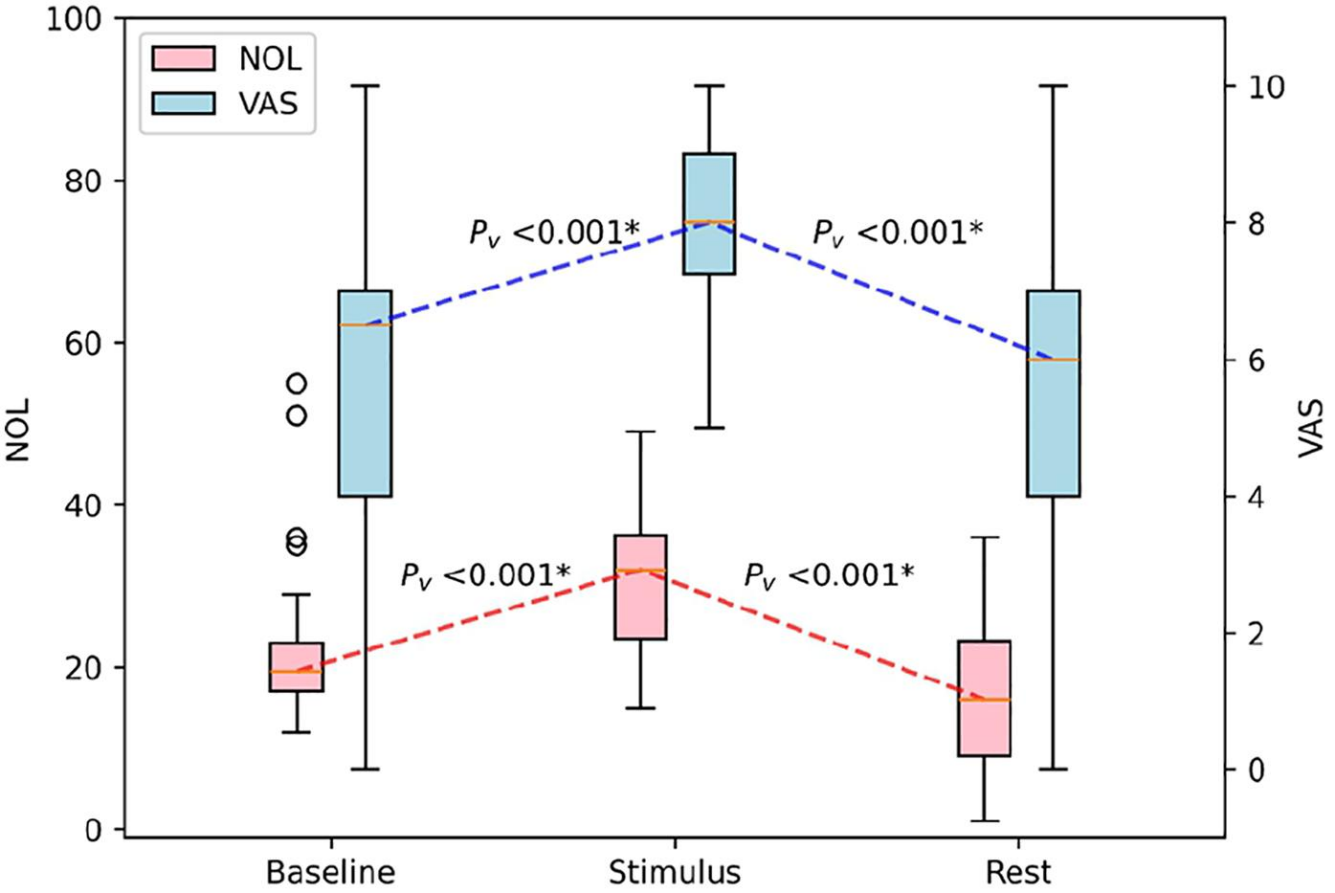
Boxplot of the impact of physiotherapeutic exercise on NOL and VAS scores in patients with CRPS. NOL, nociception level index (0–100); VAS, Visual Analog Scale (0–10, where 0: no pain and 10: worst imaginable pain), * statistically significant with *p* < 0.00625.

Before initiating physiotherapy exercises, participants reported moderate pain levels, reflected by an average VAS score of 5.5 ± 2.8. The NOL score was recorded at 22.7 ± 10.7. During the exercise itself, both VAS and NOL scores increased. Subjective pain, as measured by the VAS, increased to 8.3 ± 1.5, while objective pain, measured by the NOL index, increased to 30.3 ± 8.9 (Table 2). This change in scores was statistically significant (*p* < 0.001) (Figure 2).

**Table 2:** NOL and VAS values at different time points in the study.

Summary	NOL	VAS
Baseline		
n	26	26
Mean (SD)	22.65 (10.69)	5.46 (2.77)
Median (Q1-Q3)	19.50 (17.00–23.00)	6.50 (4.00–7.00)
Min, Max	12.00,55.00	0.00,10.00
Median 95% CI	17.50,22.00	4.00,7.00
1st physiotherapy		
n	26	26
Mean (SD)	30.27 (8.88)	8.15 (1.57)
Median (Q1-Q3)	32.00 (23.50–36.25)	8.00 (7.25–9.00)
Min, Max	15.00,49.00	5.00,10.00
Median 95% CI	26.00,34.00	8.00,9.00
Rest after 1st physiotherapy		
n	26	26
Mean (SD)	16.42 (9.19)	5.23 (2.86)
Median (Q1-Q3)	16.00 (9.00–23.25)	6.00 (4.00–7.00)
Min, Max	1.00,36.00	0.00,10.00
Median 95% CI	12.00,20.50	4.00,7.00
2nd physiotherapy		
n	26	26
Mean (SD)	35.27 (12.23)	7.50 (2.16)
Median (Q1-Q3)	34.00 (28.50–44.00)	8.00 (6.00–9.75)
Min, Max	8.00,60.00	3.00,10.00
Median 95% CI	30.50,43.00	6.00,8.51
Rest after 2nd physiotherapy		
n	19	19
Mean (SD)	29.42 (15.59)	4.68 (2.75)
Median (Q1-Q3)	29.00 (21.50–38.50)	4.00 (3.00–7.00)
Min, Max Median 95% CI	3.00,59.00 25.00,31.00	0.00,10.00 3.00,6.00

NOL and VAS values at different time points in the experiment.

CI, confidence interval; NOL, nociception level index; Q, quartile; SD, standard deviation; VAS, visual analogue scale.

### During a 2 h lidocaine treatment

As shown in Table 2, before the administration of lidocaine, while at rest and calm, participants reported a baseline VAS of 5.2 ± 2.8 and the NOL index indicated a resting state value of 16.4 ± 9.2. Both the VAS and NOL scores decreased respectively following the rest period. Throughout the two-hour intravenous lidocaine infusion period, participants were asked every 20 min regarding their perceived pain intensity, with a concurrent record of NOL index values. The mean (±SD) VAS scores recorded from the first to the sixth measurements were as follows: 5.5 ± 2.9, 6.0 ± 2.9, 5.7 ± 2.9, 5.4 ± 2.8, 5.1 ± 2.6, and 4.5 ± 3.2, respectively. Corresponding mean (±SD) NOL index values were recorded at 20.7 ± 10.6, 26.4 ± 12.3, 27.0 ± 14.5, 23.5 ± 14.8, 24.1 ± 13.2, and 22.6 ± 10.8, respectively.

### Post-Lidocaine treatment: pre- and post-physiotherapy exercise

Following this two-hour lidocaine administration, a physiotherapeutic exercise was again conducted, resulting in a reported VAS score of 7.5 ± 2.2, with NOL index values increasing accordingly to 35.3 ± 12.2. This increase was significant compared to the resting values recorded before the exercise (*p* = 0.009).

**Figure 3:**
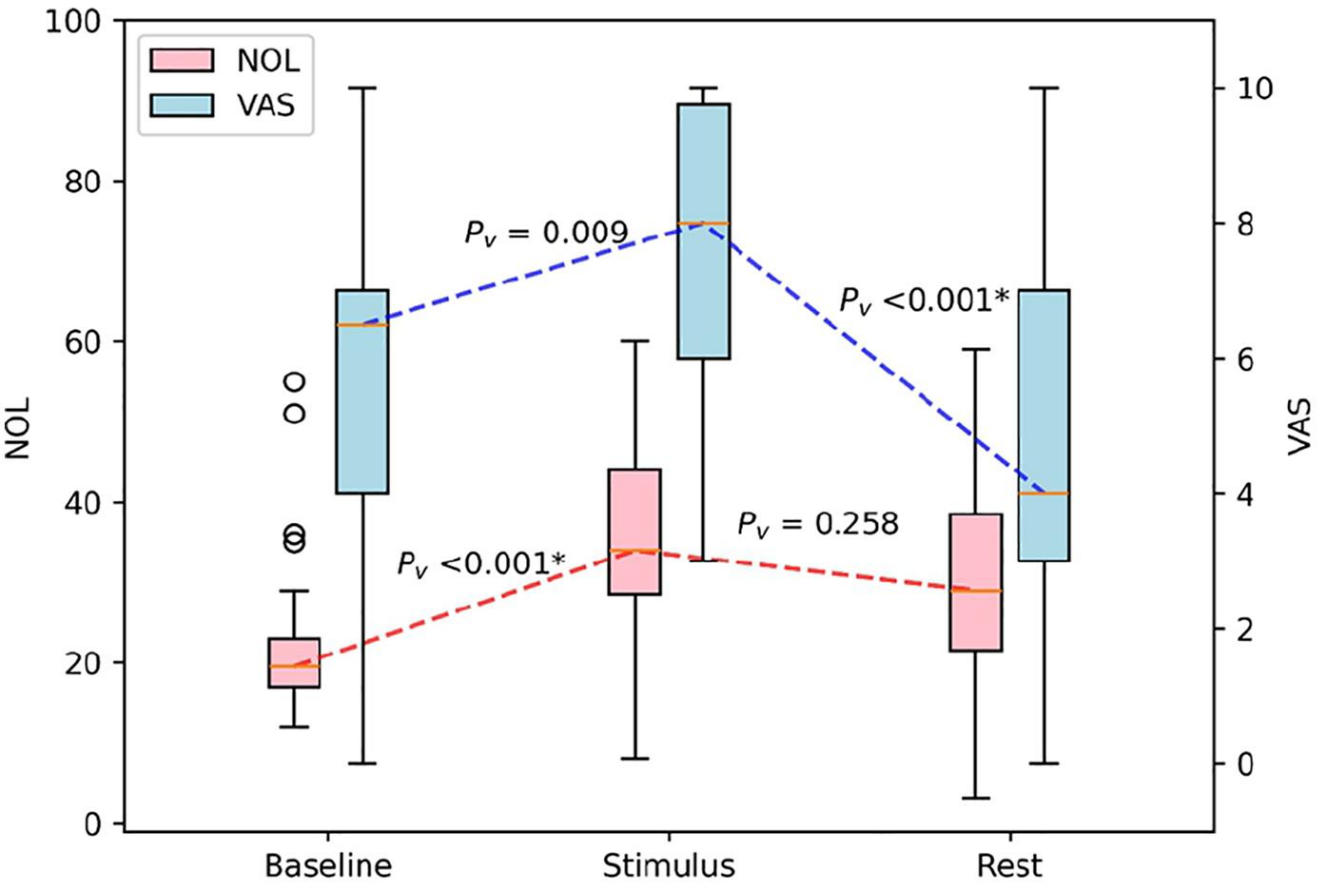
Boxplot of the impact of physiotherapeutic exercise after lidocaine treatment on NOL and VAS scores in patients with CRPS. NOL, nociception level index (0–100); VAS, Visual Analog Scale (0–10, where 0: no pain and 10: worst imaginable pain), *statistically significant with *p* < 0.00625.

During the rest period, post-physiotherapeutic exercise VAS scores were significantly lower and NOL index values trended towards lower scores after lidocaine treatment, compared to the previous physiotherapeutic exercise period. The VAS score decreased to 4.6 ± 2.7 and the NOL index to 29.4 ± 15.6 (*p* < 0.001, *p* = 0.258, Figure 3).

### Receiver operating characteristic curve

**Figure 4:**
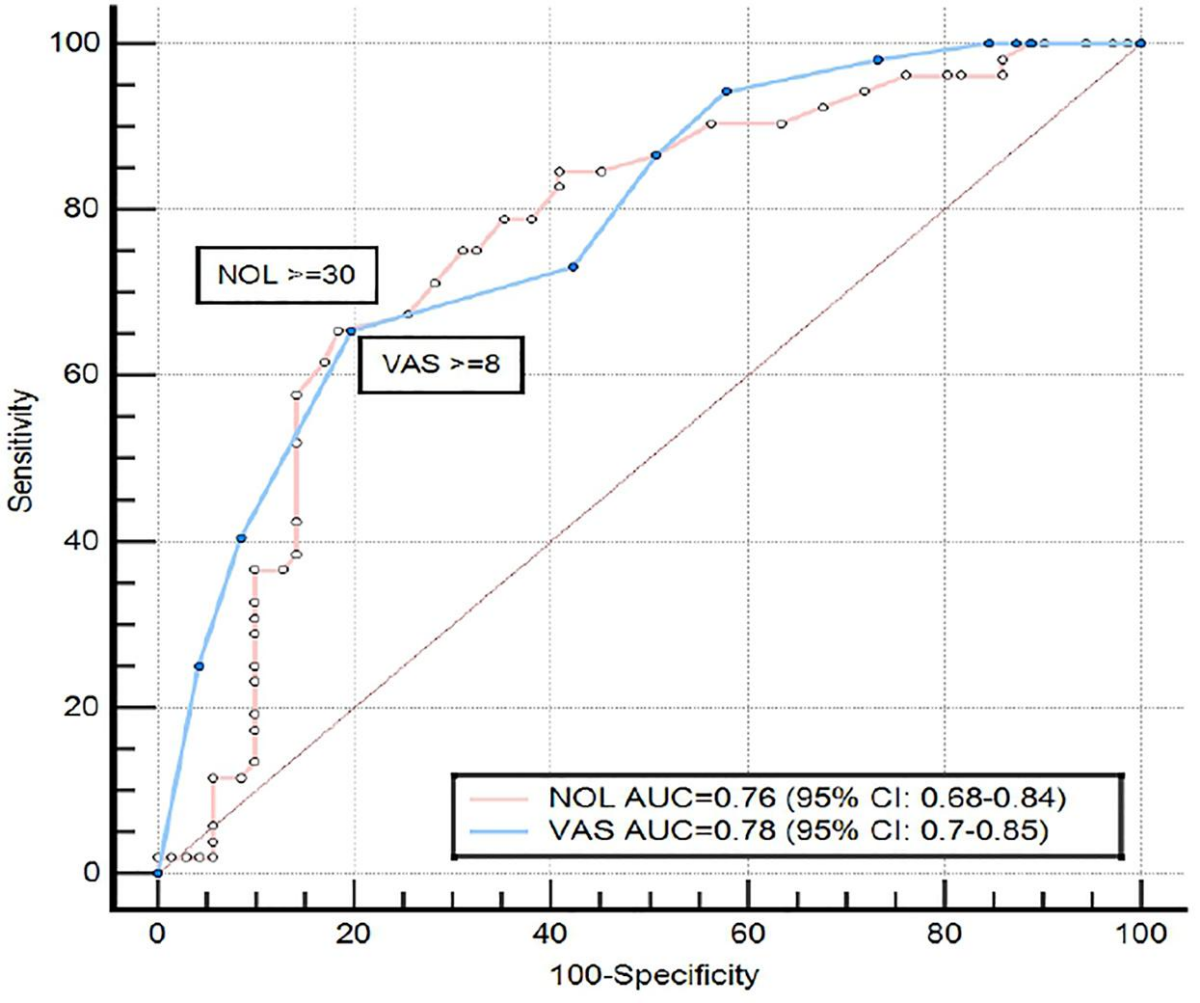
Receiver operating characteristic curve, comparing the ability of NOL and VAS to discriminate between painful stimulus and no stimulus in patients with CRPS. The maximal Youden's Index value of the NOL ROC curve was reached at a specificity of 81.7% and sensitivity of 65.38%, and the maximal Youden's Index value of the VAS ROC curve was reached at a specificity of 80.3% and sensitivity of 65.39%. NOL, nociception level index (0–100); VAS, Visual Analog Scale (0–10, where 0: no pain and 10: worst imaginable pain; CRPS, complex regional pain syndrome; AUC, area under the curve; CI, confidence interval).

Finally, ROC curve analysis for sensitivity and specificity of each parameter (NOL index, and VAS score) to detect the pain induced by the experimental stimulus, demonstrated that NOL had the same sensitivity and specificity as VAS score: AUC NOL 0.76, (95% CI: 0.68–0.84), vs. AUC VAS 0.78, (95% CI: 0.70–0.85) (Figure 4).

## Discussion

Pain assessment remains a critical aspect of clinical care, influencing treatment decisions and patient outcomes. The International Association for the Study of Pain (IASP) defines pain as “an unpleasant sensory and emotional experience associated with, or resembling that associated with, actual or potential tissue damage” (9). This definition underscores the subjective nature of pain, emphasizing that pain is fundamentally what the patient experiences and reports.

Traditional methods such as the Visual Analog Scale (VAS) and Numeric Rating Scale (NRS) provide subjective assessments based on patient self-reporting. These tools are widely used and valuable, particularly in acute pain settings. However, they may have limitations in chronic pain contexts, where pain perception can be significantly influenced by psychological factors, emotional states, and the chronicity of the condition (1, 10).

The subjective nature of pain assessment presents challenges in clinical practice. Patients' self-assessments may sometimes be questioned or misinterpreted, leading to issues of over- or under-reporting of pain. This can result in suboptimal pain management, as clinicians may struggle to accurately gauge the severity of a patient's pain or the effectiveness of prescribed treatments (11).

Moreover, the intermittent nature of these subjective assessments fails to capture the dynamic and fluctuating nature of pain experiences. Clinicians often lack continuous, real-time data on pain levels, which could provide valuable insights into pain patterns and responses to interventions.

These challenges highlight the need for more objective, continuous measures of pain and nociception. Such tools could complement patient self-reports, providing clinicians with additional data to inform their decision-making processes. Objective measures could help validate patient experiences, potentially improving the accuracy of pain assessments and the effectiveness of pain management strategies (12).

By integrating both subjective patient reports and objective physiological measures, clinicians may be better equipped to understand and address the complex, multifaceted nature of pain experiences. This approach could lead to more personalized and effective pain management strategies, ultimately improving patient outcomes and quality of life (13). Objective measures like the NOL index offer potential advantages by integrating physiological parameters to quantify pain responses (13–15). The major findings in this study suggested a notable difference for only some of the patients' self-reported pain levels using the VAS and the objective measurements of autonomic responses via the NOL index, as well as comparable discriminative ability in detecting pain-related responses, demonstrated by similar AUC values. These findings highlight a potential utility of the NOL index as a practical tool for identifying pain in patients with cognitive or language impairments, where self-reporting is limited.

Participants reported moderate pain levels upon study entry, averaging 5.46 (SD 2.77) on the VAS. This indicates ongoing pain at the time of assessment. In contrast, the baseline NOL index showed a relatively stable mean of 22.65 (SD 10.69). This discrepancy suggests several factors: Firstly, the clinical environment in which pain assessments are conducted may influence how patients perceive and report their pain levels. Factors such as the presence of healthcare providers, the anticipation of assessments, and the overall setting of a clinical visit can all impact subjective pain reporting. Secondly, psychological factors are known to play a significant role in chronic pain conditions like CRPS (16). Conditions such as anxiety, depression, and fear of pain can influence how individuals subjectively experience and report their pain levels. These psychological factors may not necessarily correlate with changes in autonomic responses measured by the NOL index.

In clinical practice, a painful physiotherapy exercise involving the affected limb was preferred over a standardized painful stimulus due to its greater clinical relevance. The primary aim in managing pain in patients with CRPS and other similar conditions is to promote rehabilitation and improve movement, strength, and functional outcomes (12, 17, 18). Assessing pain within a dynamic, contextually meaningful framework holds more clinical significance than in static, resting conditions. However, the lack of standardization in the exercise protocol introduces variability in pain intensity among patients.

Following exposure to the initial painful stimulus, both Visual Analog Scale (VAS) scores and Nociception Level (NOL) index values increased significantly. Mean VAS scores rose to 8.15 (SD 1.57), indicating a substantial increase in perceived pain intensity. Similarly, the NOL index showed a significant elevation to a mean of 30.27 (SD 8.88), reflecting heightened autonomic responses to nociceptive stimuli. This simultaneous increase highlights the relationship between subjective pain perception and objective autonomic activation during acute pain experiences.

Lidocaine is a well-established and widely accepted treatment for pain management in CRPS. Its use is standard practice in many pain management centers, including Carmel Medical Center, due to its proven efficacy in alleviating neuropathic pain symptoms associated with CRPS. The mechanism of action involves blocking sodium channels in nerve fibers, which helps to reduce pain signal transmission. In our study, we studied the effects of lidocaine using the NOL index and VAS scores to assess changes in pain perception. Following lidocaine administration, we assessed significant increases in both the NOL index [35.27 (SD 12.23)] and VAS scores [7.50 (SD 2.16)] after the second exercise session, indicating that this session was still very painful. This observation prompts consideration of several factors: firstly, lidocaine may have provided inadequate pain relief, potentially due to being administered at an initial, possibly subclinical dose as per department protocol of first treatment, with gradual lidocaine increase in follow up treatments that were not part of the study. Secondly, the high pain perception could also be influenced by the underlying complexity of CRPS pain mechanisms, which may not fully respond to lidocaine alone. These findings underscore the complex nature of lidocaine's analgesic effects and highlight the need for further research to optimize its use in CRPS management.

This study has several limitations. First, the small sample size (*n* = 26), along with data collection loss from the second physiotherapy exercise, limits the external validity and generalizability of our findings. For this reason, and given the exploratory nature of this feasibility phase, no control arm was included. The study focused on a single cohort of CRPS patients to determine whether the NOL, previously validated in anesthetized patients, could be feasibly applied and produce interpretable readings in awake individuals. Including healthy or alternative chronic pain control groups within such a limited sample would have further reduced statistical power and compromised internal comparisons. Further future studies involving larger cohorts are necessary to comprehensively evaluate the NOL index in awake patients with chronic pain across various conditions, with different analgesic agents and at full therapeutic concentration. Second, the potential for non-pain-related stimuli to influence the autonomic parameters measured by the NOL index in awake patients. Unlike anesthetized patients whose physiological responses are primarily related to the surgical procedure, awake individuals experience physiological fluctuations unrelated to pain that can confound the interpretation of the NOL index. These confounding factors include movement and communication. Even with instructions to minimize movement and talking, these activities, along with emotional stress can significantly impact autonomic parameters measured by the NOL index. Moreover, in the study only the “acute on chronic” reaction of patients with CRPS was examined, ignoring the complex cortical effect of CRPS and possible autonomic dysregulation caused by it. Future studies should explore alternative data collection methods that minimize participant movement, such as employing specialized recording equipment that isolates relevant physiological signals. Moreover, chronic pain is often comorbid with significant anxiety and stress, both of which are known to affect autonomic responses measured by the NOL index.

## Conclusion

Our study demonstrates the feasibility of the NOL index as an objective pain assessment tool, in chronic pain patients with CRPS. While the VAS provided subjective reports of pain intensity, the NOL index measured physiological parameters of the ANS, potentially offering a more objective assessment. NOL index increased significantly when patients with upper limb CRPS reported acute pain, indicating that the autonomic parameters of nociception might be used to assess acute pain in this population. These findings highlight the NOL index's potential as a valuable complementary tool for chronic pain assessment.

Future studies with a standardized lidocaine dose or with other analgesic drugs or techniques could further clarify the NOL index's effectiveness compared to subjective scales, particularly when analgesics are involved. This would strengthen the evidence for the NOL index's role in guiding treatment decisions and improving patient outcomes in chronic pain management.

## Data Availability

The raw data supporting the conclusions of this article will be made available by the authors, without undue reservation.
